# Iodine and Pregnancy—A Qualitative Study Focusing on Dietary Guidance and Information

**DOI:** 10.3390/nu10040408

**Published:** 2018-03-26

**Authors:** Maria Bouga, Michael E. J. Lean, Emilie Combet

**Affiliations:** Human Nutrition, School of Medicine, College of Medical, Veterinary and Life Sciences, 10–16 Alexandra Parade, University of Glasgow, Glasgow G31 2ER, UK; mairabouga@gmail.com (M.B.); mike.lean@glasgow.ac.uk (M.E.J.L.)

**Keywords:** iodine, pregnancy, qualitative research, awareness, perceptions, nutrition

## Abstract

Iodine is essential for thyroid hormones synthesis and normal neurodevelopment; however, ~60% of pregnant women do not meet the WHO (World Health Organization) recommended intake. Using a qualitative design, we explored the perceptions, awareness, and experiences of pregnancy nutrition, focusing on iodine. Women in the perinatal period (*n* = 48) were interviewed and filled in a food frequency questionnaire for iodine. Almost all participants achieved the recommended 150 μg/day intake for non-pregnant adults (99%), but only 81% met the increased demands of pregnancy (250 μg/day). Most were unaware of the importance, sources of iodine, and recommendations for iodine intake. Attitudes toward dairy products consumption were positive (e.g., helps with heartburn; easy to increase). Increased fish consumption was considered less achievable, with barriers around taste, smell, heartburn, and morning sickness. Community midwives were the main recognised provider of dietary advice. The dietary advice received focused most often on multivitamin supplements rather than food sources. Analysis highlighted a clear theme of commitment to change behaviour, motivated by pregnancy, with a desired focus on user-friendly documentation and continued involvement of the health services. The study highlights the importance of redirecting advice on dietary requirements in pregnancy and offers practical suggestions from women in the perinatal period as the main stakeholder group.

## 1. Introduction

Iodine is key for synthesis of the thyroid hormones, which play a vital role in normal brain development in fetal and postnatal life. Iodine deficiency (ID) during pregnancy (defined as a median population urinary iodine concentration (UIC) of less than 150 μg/L) or neonatal life (<100 μg/L) [1] is the most preventable cause of brain retardation for the infant [2]. The consequences of ID range from subtle loss of intelligence quotient (IQ) to cretinism. Recent evidence indicates that there is mild iodine insufficiency in the United Kingdom (UK) and several other European countries (Finland, Italy, Latvia), with limited insight into the consequences of this insufficiency for children’s cognitive development (e.g., reduced verbal IQ, poorer educational attainment, reduced reading speed) [3,4].

ID is a global issue [2], not limited to developing countries or high-altitude areas where endemic cretinism was classically found. Based on the 2017 International Council for the Control of Iodine Deficiency Disorders (ICCIDD) global map and scorecard of ID, Europe has a high number of countries where iodine intakes are of concern, including Denmark, Estonia, Finland, Ireland, Italy, Lithuania, and the UK. Women in the UK have been shown, in national and sub-national studies, to be iodine insufficient at a population level [5,6,7]. The UK Reference Nutrient Intake for adults is 140 μg/day and the recommended level of intake according to the WHO/United Nations Children’s Fund (UNICEF)/ICCIDD is 150 μg/day [2], which is not met by schoolgirls in the UK (68% below threshold) [7]. After conception, the WHO recommendation rises to 250 μg/day, which is only met by 40% of pregnant women [8]. The European Food Safety Authority (EFSA) recently proposed a new reference value for adequate intake for pregnancy (200 μg/day). In the UK, there is no proposed increment for iodine intake during pregnancy and lactation. 

Although recommendations focus on daily consumption, adequate status may also be achieved through intermittently greater intakes, to reach an average intake that meets the recommended intake. Iodine is stored in the thyroid (~75% of the total body iodine −15–20 mg), and daily usage from the thyroid store is estimated to 60–80 μg in non-pregnant adults [9]. 

The main dietary sources of iodine in the UK are dairy and seafoods, which together make up 13% of energy intake in adult women [10]. Recommended intakes could potentially be met by consuming two portions of white sea fish per week, in addition to the equivalent of two glasses of milk (as drinks, or in cereals for example), a yoghurt, and a cheese serving per day. However, many women avoid these foods and lack knowledge and know-how to include them in their diet. Including milk in the diet is the factor that contributes most toward iodine status, as found in a Danish study with more than 4500 participants [11]. Rasmussen et al. [11] showed that the risk of a low iodine intake is higher in those whose diets do not include at least 0.5 L milk per day and 200 g of fish per week. Stricter forms of the vegetarian diet, which excludes fish and seafood products consumption, are also associated with a higher risk of iodine insufficiency, based on measured urinary iodine excretion [12]. Although seaweed is an acceptable food for vegetarians, it is not widely consumed, and 25% of vegetarians and 80% of vegans have an insufficient iodine status compared to 9% of non-vegetarians [13,14]. Vegans also have been shown to have a lower urinary iodine excretion compared to vegetarians (78.5 μg/L vs. 147 μg/L, *p* < 0.01) [15].

The consequences of an iodine insufficient population include personal and societal costs. The absence of prophylactic fortification in the UK, combined with the low knowledge and awareness of iodine nutrition, has led to iodine insufficiency becoming a serious public health concern. In a recent meta-analysis, ID children aged five years and under had a 6.9 to 10.2 lower IQ compared to iodine replete children [16]. In the Mother and Child Cohort Study (MoBa) study, a recent large prospective cohort study in Norway, children born from mothers who consumed insufficient iodine from foods during pregnancy have significantly higher attention-deficit/hyperactivity disorder symptom scores [17]. The cost effectiveness of iodine supplementation in pregnancy has been modelled, suggesting a saving of £199 in healthcare costs and £4476 societal costs for an increase of 1.22 IQ points per offspring [18]. 

There is a sustained debate on the ethical implications of a randomised controlled trial of iodine supplementation in pregnancy, in parallel with concerns over the adverse effects of salt and the conflicted message that salt iodisation would convey [19,20]. There are a range of other strategies to tackle iodine insufficiency, including updating dietary recommendations, introducing mandatory salt fortification, and new nutritional education strategies aiming to increase awareness and promote iodine rich foods [21,22]. In a UK survey, over half of mothers (55%) could not identify correct sources of iodine, commonly mistaking salt (21%) and vegetables (54%) as iodine-rich foods. Moreover, healthcare professionals could not recognise iodine-rich foods either [23]. Most women (87%) reported willingness to modify their dietary behavior, if they received information related to the importance of iodine in pregnancy [8].

To find an effective way to address iodine insufficiency, there is a need to explore the current level of awareness about iodine in pregnancy and related dietary recommendations. Few studies in the UK have explored the level of knowledge and awareness of iodine [8,24] and highlighted the need to understand in-depth the perceptions of women in relation to dietary guidance, the way these are provided, and the most endorsed approach for provision of such recommendations.

This qualitative study is articulated around three main research questions, engaging stakeholders to canvass current perceptions and experiences of dietary guidance and recommendations relating to iodine in pregnancy:

1. What is the current perceived level and quality of dietary guidance received by expectant mothers and new mothers?
2. What are the perceived barriers to increasing or maintaining an adequate intake of dairy and seafood pre-conception and during pregnancy/lactation?
3. What would be the most effective delivery of dietary guidance to expectant and new mothers?

## 2. Materials and Methods 

### 2.1. Study Design and Choice of Methods

A cross-sectional design was used to explore stakeholders’ experiences, views, and attitudes. Participants’ perceptions, opinions, beliefs, and attitudes were captured through interviews, either face-to-face or by phone. Topic guides included elements relevant to the Health Belief Model [25]. A food frequency questionnaire (FFQ) (validated for iodine-rich food intake [26]) was used to explore demographic characteristics, iodine intake, and practices related to pregnancy. The qualitative approach was chosen as it provides a better understanding and allows the exploration of issues that have not been deeply investigated by existing quantitative research [27]. It aims to give voice to people talking about their beliefs and expectations, instead of focusing on a range of pre-determined questions in semi-quantitative surveys [28].

### 2.2. Subjects

Recruitment took place from May to December 2015 in a community setting, by snowball sampling. Women were recruited through social media, fora, online advertisement, and by word of mouth. Participants were provided with the study information and gave informed consent.

The inclusion criteria were English-speaking women (fluency level sufficient to follow an active conversation), of childbearing age, living in the UK, having a baby (younger than two years old), being pregnant, or planning to start a family. There were no further restrictions in terms of selection. 

### 2.3. Questionnaire

Socio-demographic characteristics were recorded in the first part of the questionnaire, including smoking status (those answering “yes” to the question “Are you currently a smoker?” were categorised as smokers), use of medication, and self-reported anthropometric measures (pre-pregnancy weight, current weight, height). Education level was categorised as school level (School certificate, standard grade/GCSE (General Certificate of Secondary Education), Highers/A levels), college level (HND (Higher National Diplomas)/HNC (Higher National Certificates), NVQs (National Vocational Qualifications), other higher education diploma), undergraduate degree (Bachelor degree), and postgraduate degree (Master’s Degree, Ph.D.). To assess iodine intake with minimal participant burden, we used our previously validated short FFQ which captures the intake frequencies of iodine-rich foods in eight categories (i.e., milk, oil-rich fish, white sea fish, other seafood, cheese (hard and soft), yoghurts, milk or cream-based puddings, cheese-based dishes) [26]. Participants were categorised according to their iodine needs: participants with “increased demands” (250 μg/day, pregnant and breastfeeding) and “basic demands” (normal adult demands, 150 μg/day). Questions on nutrients’ awareness, iodine confidence, and dietary changes during pregnancy (increased, decreased, or maintained intake of a list of foods) were also included as part of the questionnaire, as previously described [8].

### 2.4. Interviews

All interviews were conducted by the same researcher either in person or over the phone. Participants were already in possession of the study information sheet and had provided informed consent. Prior to the start of the interview, the overall process was explained again, and participants were notified that interviews would get recorded.

A narrative focusing on current barriers to adequate dairy, seafood, and ultimately iodine intake, as well as desired content and mode of delivery for dietary guidance/recommendation, was obtained through interviews with the recruited participants. Outcome measures were qualitative and analysed using thematic analysis. 

Interviews were structured and followed a topic guide based around the Health Belief Model:

1. Form of dietary guidance received before pregnancy/during pregnancy/lactation.
2. The role of received dietary guidance in shaping food choices.
3. The perceived recommended levels of intake for iodine in pregnancy.
4. Knowledge on how the recommended intake of 250 μg per day iodine in pregnancy can be met.
5. Barriers and facilitators in meeting an adequate intake of dairy and seafood in pre-conception and during pregnancy and lactation.
6. Opinions on the best way to deliver dietary recommendations which are understandable and practical, regarding iodine nutrition.

As part of the interview process, participants were given coloured photos of different foods (dairy products, milk, salt, red meat, sushi, vegetables, fish and seafood) and were asked to name the sources of iodine. They were also provided with pictures of iodine-rich food portions (a glass of milk, milk in drinks, a portion of cheese, a pot of yoghurt, portions of fish), to estimate a combination that would cover their requirement for iodine in pregnancy.

The interview topic guide was pre-tested for clarity and comprehension in a group of women that did not take part in the main study. Validity and reliability were secured in the study during the design of the topic guide, the interviews process, and analysis, by following Yardley’s [29] principles for assessment of qualitative research (i.e., sensitivity to context, commitment and rigour, transparency and coherence, impact and importance). In every step of the study, the authors followed the criteria of quality assessment, from the level of design to the level of data presentation.

Interviews were completed after reaching saturation in the upcoming themes. Interviews stopped when a lack of novel contributions was evident; the data collected were transcribed verbatim. Transcripts were reviewed by two researchers; after agreement that data saturation was reached, study recruitment closed.

### 2.5. Data Analysis and Statistics

All quantitative data from the socio-demographics and FFQ questionnaires were entered in an SPSS database. Descriptive statistics were calculated. Parametric data were described as mean and standard deviation and non-parametric as median and interquartile range (IQR). The FFQ data were analysed according to Combet and Lean [26]. The statistical software SPSS version 21.0 (IBM Corporation, New York, NY, USA) was used. 

All interviews were audio-recorded, transcribed verbatim, and analysed with thematic analysis by following the four stages of the analysis: familiarisation with transcripts and data, generation of initial codes, searching for themes, and reviewing themes. NVivo version 11 (QSR International, Doncaster, Australia) was used in the analysis.

## 3. Results

### 3.1. Participants’ Characteristics and Awareness of Iodine Importance

Only women meeting all inclusion criteria were recruited to the study. A total of 54 women were recruited to the study, of which six failed to complete the interviews and the short questionnaire, resulting in 48 women taking part. At the time of their participation, 38% were pregnant, 35% were breastfeeding, 10% were planning to start a family or were actively trying to conceive, and 17% had a baby or a toddler (younger than two years old) but were not breastfeeding. A minority were following a vegetarian diet (*n* = 4, 8%), and 17% were obese. All participants’ characteristics are shown in Table 1. Out of the 48 interviews, 40 were phone and eight face-to-face interviews, with an average duration of 10:46 min (range 06:33–18:06 min).

Participants’ awareness of iodine was poor. Only 23% (*n* = 11) had heard about iodine, lower than for other nutrients: folic acid 100% (*n* = 48), iron 92% (*n* = 44), calcium 85% (*n* = 41), vitamin D 75% (*n* = 36), and vitamin A 63% (*n* = 30). Only 25% (*n* = 12) reported awareness of the role of iodine in the development of the unborn baby. Confidence on how to achieve adequate iodine intake during pregnancy was low (score 1, 2, or 3 in a 7-point Likert scale) in 72% (*n* = 34) of the participants (mode = 1). 

### 3.2. Iodine Intake

Out of the 48 participants, 73% (*n* = 35) had increased daily iodine demands according to WHO/UNICEF/ICCIDD and EFSA as they were pregnant or breastfeeding. Table 2 shows the median iodine intakes in those with basic or increased demands and the contributions of a range of food sources, as well as the proportion of the WHO/UNICEF/ICCIDD recommended intake achieved in each group, through diet and supplements.

Dietary change in pregnancy varied among participants, with up to 16% reporting to decrease at least one type of dairy product, 22% reporting to cut-out a type of fish or seafood product, while 40% and 16% reported increasing dairy or fish/seafood, respectively. 

### 3.3. Qualitative Results

**Figure 1:**
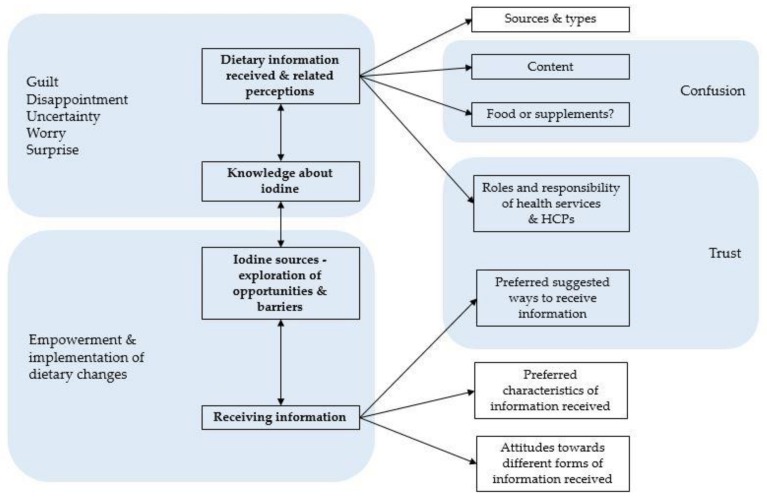
Themes and main subthemes of the analysis and their relationships. HCPs: healthcare professionals.

Five main themes emerged from the analysis of the 48 interviews, summarised in Figure 1 with associated subthemes and their inter-relationships. The first theme included views about information received during the periods of preconception and pregnancy and focused on sources, content, and form of information, as well as perceived problems related to this information and attitudes of participants towards this advice. The second theme focused on the level of participants’ iodine knowledge as a nutrient and the recommendations for pregnancy. The third theme was around the exploration of the acceptance of iodine sources and any barriers related to their intake. The fourth theme included views on preferred ways of dietary information delivery in the perinatal period of life. In parallel, the emotional dimension of receiving nutritional guidance in pregnancy emerged throughout the interviews. 

#### 3.3.1. Dietary Information Received and Related Perceptions

##### Sources and Form of Received Dietary Information

There were differences in the type of informational support that participants had received in pregnancy nutrition, by case and between UK regions. Most had received written guidance (leaflets/booklets) from community midwives, mainly during the first antenatal care appointment (IT39: “*It was really only some leaflets that I was given by the midwife. I don’t think she actually talked to me through or anything, it was just literature she handed over.*”). Those living in Scotland mentioned the “Ready Steady Baby” as a source of information about pregnancy nutrition either as a book, a website, or an application. No similar resource was mentioned by participants from other geographical areas. 

Verbal information was seldom commented on (IT14: “*My midwife on my first appointment asked whether I was aware (about dietary guidelines for pregnancy*), *I said I was generally aware but then she gave me general points.*”). Short discussions with the midwives regarding nutrition were less frequent and mainly included a brief summary of the written information, mostly after participants’ personal enquiry (IT05: “*It was I who brought it up with my midwife on one of the first visits. I had a couple of questions about how cheese and various things like that and it was me that actually brought it up with her. She didn’t bring it up with me and really she answered a couple of questions but then told me to refer to this folder I’ve been given.*”). Apart from the community midwives, who were mostly providing nutrition-related advice, general practitioners were also mentioned in providing verbal guidance, as well as friends and family. A quarter of the participants said that no one has spoken to them about nutrition in pregnancy. Participants interested in learning more about nutrition, including those that have received some form of information, did personal research, either online or using books.

##### Nutrition Information Content

Pregnancy nutrition-related information fell under three themes: eating a healthy balanced diet, avoiding certain foods, and taking folic acid and/or other multivitamin supplements. Most participants could not remember or list specific received nutrition information, and when they could, it was mainly about (i) avoiding uncooked and unpasteurised foods, alcohol, caffeine; and (ii) taking supplements. Lack of information regarding diet during lactation was also reported.

Around half of the participants highlighted how the information received during pregnancy was mainly related to the foods and other things to avoid (e.g., smoking, alcohol, raw fish and meat, vitamin A, certain types of cheese) rather than foods to increase or maintain in their diet (IT21: “*It was more about what you couldn’t eat though than what you should eat. It was more about avoiding things like caffeine and certain types of food rather than what was the best to eat.*”)*.* A group of the participants who reported having received information on what should be eaten during pregnancy believed that messages regarding foods to avoid were the most powerful.

Multiparous participants reported having received less information in subsequent pregnancies compared to their first one, as prior knowledge was assumed (IT42: “*I think I didn’t receive a lot this time round, because this is my second pregnancy I think they kind of assumed I that I knew it from before.*”).

##### Foods or Supplements?

A minor theme focused on multivitamins and folic acid with little information or knowledge on the foods that contain the required micronutrients (IT15: “*When I was told in the beginning from my midwife you shouldn’t have too much vitamin A, nothing was really explained why and I am still not really clear what foods vitamin A is in, how I should avoid vitamin A.*”). Many participants reported knowing that they should be taking supplements during pregnancy (mainly folic acid, vitamin D, or a multivitamin), having received this advice from their midwife, without information about food sources of the nutrient. A suggestion made was that iodine should be included in pregnancy supplements to cover needs, especially for those who do not know how to eat a balanced diet. Worryingly, some participants taking multivitamins felt confident that the needs are covered, justifying not paying attention to their diet (IT42: “*Since I became pregnant I’ve just been taking a multivitamin designed for pregnancy, so I am hoping that kind of covers most things.*”). Only one participant reported that she forgot her supplements and was not taking them regularly, so found food recommendations easier to implement. 

##### Role and Responsibility of Health Services and Healthcare Professionals

Participants usually referred to the health services (including the midwife, the general practitioner, the obstetrician, the health services literature, tools and services) for guidance during pregnancy. There was a high expectation that the health services would provide all the required dietary information during the first (booking) appointment or an earlier appointment, through verbal discussions, leaflets, booklets and referrals to online sources, websites, and applications. Most participants received this at their first antenatal care appointment with the community midwife (around eight to twelve weeks of pregnancy). Midwives were recognised to have experience in the field of pregnancy nutrition and were usually the first port of call when pregnant women were unsure about their diet (IT37: “*I trust very much what the midwife has to say in terms of my nutrition regarding my pregnancy, because they are quite experienced in that field.*”). However, expectations were not always met, as described below (see Section 3.3.5 “Trust”).

#### 3.3.2. Knowledge about Iodine

Iodine was an unknown nutrient for most participants, with a minority linking it to infant brain development (through personal interest, reading or research, and seldom through their community midwife). Knowledge of the relation between iodine nutrition and the thyroid hormones was limited, without any deeper specific knowledge of its importance in pregnancy.

Most participants admitted not knowing the dietary sources of iodine and attempted to guess the correct iodine-rich foods from a selection of foods depicted. Most answers were chosen randomly and were often incorrect. Only a minority identified the iodine-rich foods being dairy and seafood products.

Participants were also asked to estimate the food combination that would cover requirements for iodine in pregnancy, using pictures with portions of iodine-rich foods (a glass of milk, milk in drinks such as tea, chocolate or coffee, a portion of cheese, a pot of yoghurt, a portion of white fish, and a portion of salmon). Most food combinations proposed would not have provided a sufficient iodine intake for pregnancy and lactation. The exercise triggered surprise and hesitation in the participants (evident through their expressions, pauses, and frequent change of answers). 

#### 3.3.3. Iodine Sources—Opportunities and Barriers

Attitudes towards dairy products were generally positive as half of the participants reported finding it quite easy to include or increase dairy in the diet in substantial amounts if necessary. Milk was believed to alleviate heartburn symptoms and was often craved during pregnancy (IT25: “*Milk, I probably, I had quite a lot of milk when I was pregnant, a lot of it was because of heartburn but I would say I could drink a lot of milk.*”*).* In cases of dislike, participants still tried to increase dairy consumption via yogurts and/ or cheese (IT36: “*I do not drink milk but I do take a lot of cheese and yoghurts.*”). The main barriers towards dairy consumption included taste (mainly of milk), lactose intolerance or other health conditions believed to be associated with dairy products (such as eczema), morning sickness in the first months of pregnancy, and perceptions about the unhealthfulness of cheese in products (fat, processed foods) (IT33: “*I think with cheese I try not to each too much because I think it is quite fat*…”).

Fish and seafood consumption was perceived as harder to increase or maintain during pregnancy. Less than a fifth reported finding this easy and the barriers were further explored. For most, the main barrier was a general dislike of fish and seafood products. During pregnancy, this was exacerbated by heartburn, morning sickness, and change in taste and smell. Another major cause of fish exclusion was partners’ and familial preferences, as well as lack of cooking skills and low knowledge (IT30: “*Well, I don’t eat a lot of fish probably because my husband doesn’t enjoy it and I don’t like to cook more than one meal at once.*”; IT09: “*People probably don’t have as much knowledge on how to cook fish.*”). However, even for participants with a high intention to consume fish and seafood, confusion regarding the recommendations, compounded by the worry of eating the wrong fish species, prevented the consumption of any fish and seafood type. Cost implications, low availability, and a habit of not buying or eating these products were also regularly described barriers (IT42: “*We are not in a habit of eating a lot of fish and that’s one of the things we often say to ourselves we should have a little bit more of...*”). Vegetarians did not generally intend to change habits by including fish in their diet during pregnancy.

#### 3.3.4. Receiving Dietary Information

##### Receiving Dietary Information—Attitudes towards Different Formats

The majority of views around verbal information were positive, with only a couple of participants stating not finding a discussion helpful regarding something important about pregnancy (IT48: “*If I was to go to the doctors and ask for advice there I don’t think that would be as beneficial because (…) I feel it would be quite overwhelming (…) so if the midwife mentioned something to me I would remember but wouldn’t really know too much about it so I would have to go and look into it for myself*”). During discussions with midwives, the participants recalled the opportunity to ask questions and get more in-depth knowledge regarding the importance of the advice received, even when the discussion was short. Discussion with an expert was proposed as more useful if accompanied by a leaflet, booklet, or another visual source to refer to later, as a reminder and prompt. However, it was agreed by most that such discussions rarely happen, with leaflets usually given without going through them verbally (IT05: “*So for me, the best thing for me would have been if the midwife had given me a brief overview and then maybe given me a leaflet or a web, specific web address to take away that I could then look into more depth afterwards*”).

A common theme that emerged was also around the written information received, especially leaflets and booklets. Leaflets were read by many participants, but too many were received during pregnancy and subsequently lost, thrown out, or misplaced when required (IT48: “*I was also given information in leaflet form but during pregnancy the amount of leaflets you get is unbelievable so you don’t really pay attention, well I don’t really pay attention to a lot of the hand out stuff I was given.*”). However, participants self-characterised as “old school” written information in the form of bright, colourful leaflets with pictures that catch the attention, mainly given from the midwife or combined with verbal advice, a good and practical way of receiving information about pregnancy, diet, and specific nutrients. The information expected to appear on leaflets should be “clear”, “specific”, “comprehensive”, and “straight forward”. Leaflets were perceived as useful to remind participants about a key piece of information, more practical, smaller, and easier to read compared to books. If the leaflets were not brightly designed, participants mentioned forgetting them or not reading them (IT22: “*Basically kind of brightly designed or whatever, I would definitely pay attention to.*”).

Most participants (who were often highly educated) referred to the internet as a source of information in pregnancy. Participants admitted using simple “Google” searches to obtain information about nutrition and pregnancy, and combining information from several online sources, including several websites, BabyCentre (BabyCentre^®^ L.L.C., San Francisco, CA, USA) and healthcare services websites, fora, social media and weekly emails with leaflets, booklets, and verbal advice. However, the use of social media and other online sources is not always reliable, and often prompted by excitement about the new pregnancy and the intention to do everything right (IT05: “*These days obviously everybody goes straight on the internet, and that’s what was one of the first things that I did, but the problem I think with that is that you google various phrases and it’s more luck than judgement which websites you come up on*”; IT02: “*Well the first thing I do is look on the internet, but then you cannot really rely on that.*”). Participants preferred the internet when looking for information, but recognised that a reliable source, complete with all the required information and presented by an expert, is needed (IT30: “*I like to look online and read things of a reliable source. I might use the NHS website or something similar rather than a forum. But you need to know to look, that’s the problem.*”). The combination with other sources (leaflets, discussions) was also perceived as an effective way of getting information, as well as the weekly emails that healthcare services or other reliable sources send to pregnant women (IT37: “*The emails from the NHS, once you are registered for your pregnancy are really good because you get an email like once a week or something like that (…) they are really helpful.*”). 

Mobile applications were considered an interactive way of dealing with nutritional requirements and increasing knowledge and awareness about specific dietary needs. For many participants, an application on a smartphone would be useful, easy, and innovative if they needed to get informed about a nutrient (e.g., iodine) and try to increase it in their diet. As technology is progressing and smartphones and tablets are becoming mainstream, especially in young women, participants suggested mobile applications during pregnancy and lactation to meet an information gap related to healthy eating, supplements reminder, fitness tips, and information about fetus development. Participants proposed that if the information relating to the importance of a nutrient was integrated with one of these mobile applications, or if a new specific mobile application could be developed, that would be useful, easy, and practical, especially if endorsed by the doctor or the midwife (IT25: “*You can get apps now for everything, so maybe an app that you could give out like different ideas on how you can get that nutrient you need in the right amount, maybe like meal ideas or something like that.*”; IT05: “*The best thing for me would have been if the midwife had given me a brief overview and then maybe given me a leaflet or a web, specific web address to take away that I could then look into more depth afterwards.*”)*.* Tracking dietary intake by adding or ticking a checklist of foods commonly consumed daily via a mobile application was highlighted, with a view to tailor advice on dietary needs, including directions on what to consume to increase intake of a specific nutrient. A minority disagreed with the use of mobile applications, which they found overwhelming and burdensome (search, download, usage), preferring information from a booklet, a leaflet, or a conversation with their midwife instead (IT08: “*I found the most useful just conversation really I suppose rather than apps and research cause that’s how I think you can get a bit overwhelming for pregnant mums and for first time pregnant mums.*”)*.*

##### Receiving Dietary Information—Preferred Formats and Stakeholders’ Suggestions

Half of the participants interviewed stated that the information should be easier, quicker, clearer, more practical, basic, straight forward, and easy to remember and understand. They expressed the view that if the information was presented in a more visual, bright, and colourful way, including pictures or easy infographics, charts, and associations of nutrients with foods and portion sizes, it would stand to attention and be more likely to be remembered. 

While understanding which nutrients are important in pregnancy, a gap was identified regarding portion size and the quantity of food required to obtain the correct amount of a nutrient (IT42: “*I think having something visual that kind of shows you clearly what, like what the portions equate to is really helpful*”). While some participants were confident about the nutrients of importance in pregnancy, they were unable to translate dietary requirements to a balanced meal. There was specific uncertainty on where to get nutrients from the diet, which and how much food, and the impact of different cooking methods.

Participants were asked what they consider the best way to convey or receive information about a specific nutrient. A clear majority believed that the healthcare services were best placed to deliver this advice. The midwife, general practitioner, other healthcare professionals (health visitors, nurses, and pharmacists), dieticians, and nutritionists were in most cases considered as the best sources of nutrition information. A face-to-face discussion, early in pregnancy or at the first booking appointment, or even in an antenatal class, was often suggested. Many participants proposed that a combination of this discussion alongside written and/or online information (a leaflet, a website) would be ideal—providing the choice to return to that information later for reference. The healthcare services website and emails were also suggested, as well as the internet in general (other websites, Google search, social media, fora).

For practical advice on how to increase iodine intake, some participants reported that advice from their midwife or doctor, potentially in combination with a reference to a website, a leaflet, or a booklet, would be sufficient. For others, knowledge of key nutritional focus points was also enough—with the future health of their child being an incentive to take the required steps to initiate change. Many other ideas were discussed, including iodised supplements use; iodised salt consumption; a tick sheet or a pin board with reminders for main foods, portions, and importance of iodine; internet websites; recipes or meal plans online and in books; fridge magnets and small reference cards; supermarket magazines; advertisements; food packaging information; and the use of a mobile application with relevant information. Understanding the portion sizes and the iodine content of the foods was considered important to manage an increased need in iodine during pregnancy.

When thinking innovatively, participants proposed different tools that could be designed to deliver information regarding iodine, nutrition, and pregnancy. Approximately half of the participants preferred a mobile application for their smartphone or tablet, accessible at all times, to track dietary intake and get reminders when to adapt their diet. Mobile application tailored meal plans were also suggested, as well as improved knowledge on portion sizes, recommended intakes, and needs; a comparator was mobile applications focusing on weight management. Visual prompts were identified as important for those with low literacy. Others preferred an easy, pictorial-based, and quick to refer to tool. Recipes, meal plans ideas, pictorial-based bright leaflets, and association of foods and portions with nutritional requirements were described as desirable ways of delivering information. Other proposals included TV programmes and advertisements, public health campaigns, weekly healthcare services emails, and infographics. A minority still supported a one-to-one discussion with their midwife or doctor and a referral to a reliable website, booklet, or leaflet, as sufficient sources of information.

#### 3.3.5. Emotional Dimensions of Receiving Dietary Advice and Information

##### Confusion

Confusion about the information received was often observed. Participants reported finding different pregnancy recommendations between the UK and other countries, getting different advice between different UK areas, and receiving conflicting information from different healthcare professionals (IT09: “*And then a different midwife had a completely different attitude*.”). The lack of a single reference place with comprehensive information about all nutrients was also reported (IT26: “*They should tell you exactly what you need to have, you need to have a leaflet or like a website… It should be clearer, now it is not clear at all.*”). Some recommendations were perceived as difficult to understand and implement in terms of know-how (e.g., vitamin A content of foods, recognising unpasteurised products), and overwhelming in the context of all other dietary recommendations available. 

##### Empowerment—Implementation of Dietary Changes

Analysis highlighted a theme of clear commitment to change behaviour if prompted or made aware (IT06: “*If I knew how important it was, I would increase it.*”). Almost half of the participants highlighted the fact that if they had the knowledge, the information, and the awareness about the importance of a factor during their pregnancy (in this case mainly referring to increasing their iodine intake), they would make a conscious effort to change their behaviour or practice. The intention was already high, even from the period of preconception, as participants believed that those at that stage of life want the best for their baby and themselves. Proactivity was also evident in many cases, as nutrition was a topic often brought up by participants to their midwives. Participants asked questions and ended-up looking online for advice when they did not get the level of advice they required or expected. All participants reported having read or considered the information given to them, with the majority stating that they adjusted their diet (with a minority not changing habits through belief of already achieving a balanced diet). To feel confident of meeting all nutrients’ recommended intake, multivitamin supplements were frequently adopted.

##### Trust

A trustful source of information was very important for most participants. A clear theme of trust emerged towards the health services and the healthcare professionals. The health services were very often mentioned during the interviews and were considered as the norm for the provision of trustful dietary and pregnancy advice. The most trustful and suitable advisors were “an expert”, “the midwife”, and “the general practitioner/ doctor”, as well as the staff running antenatal classes (IT47: “*I think probably is verbally so that we could ask questions rather than just written information, and maybe not necessarily one to one but along with those pre-antenatal type classes.*”)*.* However, although initial attitudes expressed towards the health services were high expectations and trust, they were followed by disappointment regarding the actual amount of advice received. Similar feelings arose towards the general practitioner surgeries that did not provide nutrition information, which had been an expectation from several participants.

The internet and smartphone applications were considered useful sources of information, but the issue of source reliability emerged. As a result, the healthcare services website or websites proposed by experts were the online sources that participants trusted most. Moreover, the health services application and the BabyCentre application (BabyCentre^®^ L.L.C., San Francisco, CA, USA) were widely used as reliable sources.

##### Negative Feelings

Pregnancy is a stage of life when the fetus totally depends on its mother for the supply of nutrients—a fact appreciated by most participants. For this reason, participants were usually positive in making dietary and lifestyle changes (as mentioned above in “*Empowerment*—*implementation of dietary changes*”), but at the same time, this responsibility triggered negative feelings. Apart from the disappointment mentioned above, uncertainty regarding the dietary recommendations was expressed. This triggered, in some cases, worry and lack of confidence on adequate adherence to guidance (example of fish recommendations, as mentioned in Section 3.3.3.)

Towards the end of the interview, the importance of iodine for fetal development, and the richest dietary sources of iodine, were presented to the participants, alongside an indicative food combination selected to achieve the pregnancy daily recommended iodine intake. Some participants expressed surprise and disappointment at that point, and questioned why iodine had not been mentioned to them in peri-natal care. Worry and stress were also apparent through tone of voice and expressions, when participants realised the importance of iodine and were unsure whether needs had been covered (IT27: “*There is not enough guidance. As I say I felt I am quite surprised, even a little bit disappointed I didn’t know how important iodine, how important the role is it plays in the fetal development.*”). In a couple of cases, participants reported feeling overwhelmed and under pressure to follow the pregnancy recommendations, resulting in guilt for not “getting everything right” (IT08: “*That put a lot of pressure and a lot of guilt on me, but the second time round I decided to be a bit more relaxed and trust my body a bit more.*”). 

## 4. Discussion

### 4.1. Main Findings

ID is a global public health concern, with the latest ICCIDD data highlighting the general populations in as many as 20 countries, and the pregnant populations of as many as 39 countries being mildly to severely deficient [30]. Correcting ID is especially important in high risk groups, such as pregnant women and their infants. To achieve this, it is essential to understand the underlying causes of ID and the understanding of iodine nutrition by the populations concerned [22].

We explored the lack of iodine awareness and knowledge in pregnancy and the dietary and nutrition information received in and around pregnancy, to identify potential ways to improve information delivery at that stage of life. After an in-depth discussion with stakeholders, around the themes of dietary advice received in pregnancy, preferred ways of information delivery, and the knowledge and awareness of iodine nutrition, we identified gaps related to dietary advice in pregnancy and the way it is provided. Our results agree and strengthen previous findings that awareness and knowledge are low amongst pregnant women [8], even though more pregnant women achieved the WHO recommended iodine intake [2] in this sample compared to previous results (81% in this study vs. 46% in 1st trimester/40% in second and third trimesters previously [8]). Dietary guidance during pregnancy was described as confusing, focusing mostly on foods to avoid, supplements, and selected nutrients of interest (not iodine), and was not always perceived as sufficient or helpful enough to trigger effective dietary changes. Participants highlighted the need for clearer recommendations, with a clearer focus on foods and portion sizes, but also emphasised their trust in the health services. 

### 4.2. Barriers to Iodine Sufficiency

Until recently, the UK was thought to be iodine replete; however, the inadequate iodine status of the British population was highlighted in a multi-centre survey of school girls [7]. The reasons behind the re-emergence of iodine insufficiency are multifaceted and need to be explored systematically.

Most countries have implemented mandatory salt iodisation in table and food industry salt [31]. In the UK, the regulatory and legislative framework means that salt iodisation is voluntary. Iodised salt availability is low [32], and mandatory iodine fortification is still perceived to conflict with the public health message for salt reduction and chronic diseases [33]. In our study, a single participant used iodised salt and 60% reported rarely or never using salt at the table—meaning that voluntary fortification is not likely to effectively address iodine insufficiency through the consumption of table salt alone, since most sodium is consumed through ready-meals and processed foods. Universal salt iodisation has been successful in correcting ID in population level worldwide. However, studies in Italy [34], Turkey [35], and Tasmania [36] showed that ID in pregnant women persisted after the application of universal salt iodisation and awareness and knowledge of the female population regarding iodine’s role and sources remained poor after salt fortification [37]. Iodine reference intake for pregnancy has not been reviewed by the UK Department of Health since 1991, and remains similar to those of non-pregnant adults—a possible contributor to the lack of awareness. Iodine is also often lacking from pregnancy supplements and dietary guidance does not specifically cover the importance of iodine or its dietary sources. 

Pregnant women depend on community midwives during antenatal care. The health services are the main providers of dietary and lifestyle advice in pregnancy, in agreement with previous findings from Australia [38]. Consequently, dietary guidance starts around the 10th week of pregnancy, possibly too late for addressing the impact of any iodine insufficiency on fetal neurodevelopment, as the myelination process is complete in the first trimester. Our results highlight the role and importance of the health services in the provision of dietary information around pregnancy. Two contrasting feelings—one of trust, the other of disappointment, were apparent in respect to the relationship with the healthcare professionals and the delivery of information on nutrition and iodine. Awareness and knowledge regarding iodine nutrition in pregnancy have been previously found to be low amongst healthcare professionals in the UK [39] and other countries [23,40,41,42,43], and may partially contribute toward the shift from trust to disappointment. The lack of nutrition education that midwives receive is an important area to tackle [44,45], while recognising the stresses and burdens experienced by this group of healthcare professionals, especially the restricted time to cover several complex aspects of pregnancy care and related advice. 

As healthcare professionals lack nutritional knowledge, the iodine and nutrition information pregnant women receive is limited, fostering poor knowledge and awareness [8]. We highlighted divergent experiences of receiving dietary guidance in pregnancy (depth and breadth), with variations between healthcare teams, geographical areas, and parity, but also personal interest of the pregnant women, previous pregnancy experience, and educational background. Our findings confirm our previous results, showing that although information on pregnancy guidelines comes from several sources, confusion and uncertainty prevail, under the need of a reference source of reliable information. The way dietary guidance was delivered during pregnancy, and the level of depth covered, also varies between geographical areas in the UK, with inconsistencies amongst antenatal care teams, and is largely influenced by the personal interest of the pregnant women, their previous experience of pregnancy, and their educational background. As a result, our study highlights a clear need for a trustful, more comprehensive source of dietary advice in pregnancy for women to follow without feeling confused by different conflicting or unclear guidelines.

An important aspect in the landscape of strategies to address iodine insufficiency is the availability and choice of iodine-rich foods (dairy products, fish and seafood, including seaweed, iodised salt) and the potential barriers to their consumption. Since the 1970s, the demand for milk and milk products has decreased steadily [46], but barriers to their consumption are often overlooked. In an area without known ID (Sabadell, Catalonia, Spain), the iodine status of 600 pregnant women was found to be inadequate, based on their UIC (104 μg/L). The FFQ results associated milk and supplements intake with a 41% and 78% lower risk, respectively, of having UIC levels below 150 μg/L [47]. In this study, we highlight the organoleptic and behavioural dimensions linked to milk and other dairy products avoidance, including taste and smell, and, less often, adherence to a strict vegetarian or vegan diet, or its association with health-related issues (perceived or diagnose lactose intolerance). Other studies which have explored drivers of dairy food choices also identified family members’ taste preferences and needs, cost and health benefits [48,49,50], but also gender, age, and socioeconomic status [51]. Choice behaviours regarding milk and dairy products in the context of iodine nutrition are compounded by environmental factors: milk iodine concentration is lower in the spring and summer and in organic milk products [52,53]; therefore, seasonality and milk type should be taken into consideration when making recommendations and measuring intake [54], as milk remains the main source of iodine in the UK [10].

Attitudes were generally more negative towards fish and seafood products. Main barriers included general dislike due to taste and smell, followed by family’s preferences, lack of cooking skills, cost, and availability, in agreement with previous results [55]. However, an important barrier for fish and seafood consumption in pregnancy lay in the confusion in the dietary recommendations in pregnancy, namely restricting the intake of certain fish species due to heavy metals, toxins, and bacterial risk. In extreme cases, this confusion and perception of a risk led to total avoidance of fish, despite two portions of fish per week (one of which oil-rich) being advised [56]. This is consistent with the findings of Bloomingdale et al. in Boston, who found that women would rather exclude fish intake in pregnancy than risk harming themselves or their infant’s health [57].

Even though dietary information received during pregnancy is plenty, the way this information is delivered is a key factor in facilitating their implementation (including memorisation, understanding, behaviour change). Clear information, with more focus on portion sizes and foods rather than nutrients, are some of the main characteristics desired by participants. With 90% of people aged 16–24 and 87% of those aged 25–34 owning a smartphone in the UK in 2015 [58], technologically-based information delivery should be reinforced. However, the use of technology can alienate minorities (e.g., women of low socioeconomic status, low education, homeless, socially deprived, urban migrant groups) and care should be taken to avoid exclusion of those groups from accessing information, since they are often the groups needing advice the most [59]. Although technology usage, broadly, and internet access is increasing, the quality of information available online is often questionable, and quality control is needed [60]. The BabyCentre (BabyCentre^®^ L.L.C., San Francisco, CA, USA) website and application, for example, belong to the Johnson & Johnson group, a commercial entity, which participants still trusted despite the lack of affiliation to the health services. Overall, health information technology can be effective for preventive health and increasing adherence to guidelines, but cost-effectiveness data are limited and inconclusive [61].

### 4.3. Strengths and Limitations

The choice of qualitative design helped in the deep investigation of the topic of iodine nutrition in pregnancy and perceptions on the way dietary guidance is delivered in the perinatal period of life. This deep understanding of the reasons why iodine insufficiency is present in those life stages is needed to effectively address this public health issue. 

Iodine intake was quantified with our previously validated iodine specific FFQ for women of childbearing age [26], as a suitable way to classify participants based on their habitual intake of micronutrients, giving us the opportunity to know whether the perceptions of the study population would represent women with a range of different levels of intake.

Recruitment mainly took place online, to reach a population across the UK. As a result, women residing in England, Scotland, Wales, and Northern Ireland were recruited, increasing the sample’s representativeness. The study population is highly educated, with less than 30% of the participants not having a Bachelor’s degree or higher. This is however in agreement with the national statistics, as the level of education improves with the years and is higher in females of that age (childbearing age) compared to males. The 16 to 24 year old individuals Not in Education, Employment, or Training in 2014–2015 have decreased by three points since 2010–2011, accounting for only 15.3% in females of that age [62]. Comparing the study population with the British population, the education level is higher but worryingly, even in that educated young population which could also be biased in terms of interest in pregnancy nutrition, the lack of awareness regarding iodine nutrition in pregnancy remains low. 

Our sample also did not include a high percentage of obese women (17% versus 26% in the UK [63]) or smokers (1% versus 14.1% of women in the UK [64]), and had no drug or alcohol dependent women, but included 13% of non-British women. In more vulnerable groups, it is possible that dietary advice would be supplanted by focus on areas deemed more pressing—such as tobacco or alcohol dependencies. This is a dimension not explored in this study.

Lastly, information about iodine was already in the information sheet participants had received, and the question regarding the sources of iodine was asked both in the questionnaire they filled in and during the interviews. As a result, their answers were potentially influenced by these factors. 

## 5. Conclusions

The lack of iodine fortification in the UK provides an unusual, highly appropriate, ecological terrain to study the impact of a simple food or education-based intervention to tackle iodine insufficiency and its endocrine and neurodevelopmental consequences. The approach used in this study assumed that evaluating needs and expectations of stakeholders (mothers and women planning a pregnancy or breastfeeding) would enhance the design of suitable and impactful dietary recommendations and information packages, ultimately to improve iodine sufficiency. 

Iodine nutrition is important during pregnancy and lactation, but awareness and knowledge is low amongst women of childbearing age, even with high educational attainment, and among healthcare professionals in the UK.

Dietary guidance received in pregnancy is not clear, leading to confusion over recommendations. There is a need to focus on specific foods and portion sizes, rather than nutrients, supplements, and specific recommendations that are not tethered to a balanced diet and the associated know-how, that many assume is in place (such as cooking skills and knowledge of foods). Future work should incorporate users’ input to design and implement tailored health promotion approaches. In Scotland, a nutrition education intervention in pregnancy was found to be acceptable in 16–18 year old pregnant women; however, with limited effectiveness in term of changing dietary habits [65]. There is scope to build on such an intervention to improve iodine status in pregnancy. However, it is likely that, to tackle iodine insufficiency in and around pregnancy, a multi-sprung approach will be required, tackling several different angles of the problem [22]. Mobile health tools open an existing range of opportunities for personalised health, which, combined with regulatory steps, conventional dietary advice through health care professionals, and targeted awareness campaigns, would form a comprehensive approach to the public health challenge iodine has become.

## Figures and Tables

**Table 1:** Participants’ characteristics.

Demographic Data	Mean	SD
Maternal Age (years) *n* = 48	30.8	4.3
Pregnant *n* = 18	31.6	3.5
Breastfeeding/with baby *n* = 25	31.0	4.7
Planning a pregnancy *n* = 5	27.2	3.0
Child Age (weeks) *n* = 23	39.7	24.5
	**Median**	**IQR**
Maternal BMI (kg/m^2^) ^1^	24	21–29
% WHO iodine recommendation achieved		
Increased demands (250 μg/day) (*n* = 35)	81	56–122
Basic demands (150 μg/day) (*n* = 13)	99	57–134
Total daily iodine intake (μg/day)		
Increased demands (*n* = 35)	203	140–304
Basic demands (*n* = 13)	148	85–202
	*n*	%
Ethnicity		
White Scottish	16	*33*
Other White British	26	*54*
Other ethnic groups	6	*13*
Residence		
Scotland	27	*56*
England, Wales, Northern Ireland	21	*44*
Education		
School level	3	*6*
College level	6	*13*
Undergraduate degree	24	*50*
Postgraduate degree	14	*29*
Parity		
0 (or expecting first)	15	*31*
1	27	*56*
2 or more	6	*13*
Use of supplements—all (*n = 29/48*)		
Iodised	17	*35* ^2^
Non-iodised	12	*25*
*Increased demands (n = 24/35)*		
Iodised	16	*67* ^3^
Non-iodised	8	*33*
*Basic demands (n = 5/13)*		
Iodised	1	*20* ^4^
Non-iodised	4	*80*
Smokers	1	*2*
Aware about iodine ^5^	11	*23*
Low iodine confidence (1–3 points) ^6^	34	*72*

^1^ For pregnant women, the BMI (body mass index) has been calculated based on the pre-pregnancy weight reported by the participants; ^2^ Proportion of women taking iodised/non-iodised supplements out of the total sample; ^3^ Proportion of women taking iodised/non-iodised supplements out of the women with increased demands; ^4^ Proportion of women taking iodised/non-iodised supplements out of the women with basic demands; ^5^ Iodine awareness was defined as positive when the answer to the question “When it comes to healthy eating in pregnancy and lactation, have you heard of, or were you informed about iodine” was “yes”; ^6^ Iodine confidence referred to confidence on how to achieve an adequate iodine intake in pregnancy and lactation and was measured with a 7-point Likert scale (1: very low confidence −7: very high confidence). SD: standard deviation, IQR: interquartile range, WHO: World Health Organization.

**Table 2:** Iodine and iodine rich foods intake in participants with increased demands (250 μg/day, pregnant and breastfeeding) and normal adult demands (150 μg/day).

	Increased Demands (*n* = 35)		Basic Demands (*n* = 13)	
	Median	IQR	Median	IQR
Milk (g/day)	200	100–500	113	11–270
Other dairy (g/day) ^1^	119	86–192	106	80–233
Fish (g/day)	39	9–65	43	0–101
Total daily iodine from dairy (μg/day)	120	90–185	121	62–146
Total daily iodine from milk (μg/day)	54	27–136	31	3–73
Total daily iodine from fish (μg/day)	21	8–31	29	0–53
% daily iodine from dairy	83	75–97	77	68–99
% daily iodine from milk	45	23–54	24	5–47
% daily iodine from fish	16	3–24	22	0–32
Total daily iodine from food (μg/day)	152	120–199	148	85–202
Total daily iodine with supplements (μg/day)—whole sample	203	140–304	148	85–202
Total daily iodine with supplements (μg/day) only in those taking supplement	299 ^2^	215–233	550 ^3^	550–550
% WHO recommendation achieved	81	56–122	99	57–134

^1^ Other dairy includes all dairy products listed in the FFQ (food frequency questionnaire) excluding milk (i.e., cheese (hard and soft), yoghurts, milk/cream-based puddings, cheese-based dishes); ^2^ *n* = 16; ^3^ *n* = 1.

## Abbreviations

The following abbreviations are used in this manuscript:

EFSA	European Food Safety Authority
FFQ	Food frequency questionnaire
HCP	Healthcare professional
ICCIDD	International Council for the Control of Iodine Deficiency Disorders
ID	Iodine deficiency
IQ	Intelligence quotient
IQR	Interquartile range
UIC	Urinary Iodine Concentration
UK	United Kingdom
UNICEF	United Nations Children’s Fund
WHO	World Health Organisation
